# Is Wetness of Soil a Cause of Phthisis?

**Published:** 1901-03-02

**Authors:** 


### IS WETNESS OF SOIL A CAUSE OF PHTHISIS?

For a long time back it has been held that a damp soil is provocative of phthisis. Dr. Bowditch, so far back as the year 1862, laid it down that a residence on or near a damp soil was one of the primal causes of consumption in Massachusetts—probably in New England, and possibly in other portions of the globe. The late Sir George (then Dr.) Buchanan, as the result of very careful investigation of the subject in regard to England, came to much the same conclusion. He was more especially concerned with inquiring into the health results of the various sanitary improvements which had been carried out in certain English towns. These had largely consisted of drainage works, and the result of the inquiry was to show that in many towns in which drying of the subsoil had been effected the mortality from consumption had very considerably declined. Nevertheless, as pointed out by Buchanan himself, there were exceptions. Some of the towns in which undoubted lowering of the ground water had taken place had failed to reduce their phthisis mortality, while others, in which hardly any change had been effected in the condition of the subsoil, had improved markedly in regard to their consumption statistics. After this Buchanan investigated the variations in the prevalence of phthisis in different districts in the counties of Surrey, Sussex, and Kent (the area in question covering 3,182 square miles and embracing a population of 1,118,372), with direct reference to geological considerations, especially to perviousness of soil and contour of land. Putting the results of the two inquiries together, he came to the conclusion that "wetness of soil is a cause of phthisis to the population living upon it," and for many years past this view has been generally accepted. Still a good many observers have doubted whether the above dictum expressed the whole truth, and they have laid especial stress upon the exceptional cases, holding that in the absence of any obvious disturbing cause such exceptions ought to invalidate the generally accepted conclusion.

Recently Dr. Arthur Newsholme[1] has discussed this question afresh. He asks: "Is there an essental relationship between wetness of soil and phthisis mortality among the population living on such soil, or is the common excess of phthisis on wet soils rather due to the fact that a lower class of the community, worse housed and worse fed, are likely to be found dwelling on a wet soil?" and he answers that "it appears probable that much of the benefit ascribed to drying the soil has been due really to other factors of improvement which commenced to operate about the same time as the former." It has to be remembered that the essential cause of phthisis, viz. the bacillus tuberculosis, has been discovered since the days when Buchanan formulated his proposition on the subject, and we have to ask how our present knowledge as to the prime cause of tuberculosis fits in with the wet-soil theory. Like overcrowding and insufficient nourishment, the wet soil, he says, must be placed among predisposing causes; and it must be placed, furthermore, in a much lower position than the above-mentioned causes. Wet soil implies greater loss of heat by evaporation, more easy provocation of catarrhs, especially when, as would commonly happen, it is associated with cold and wet houses. But a house even on a wet soil can be effectually protected, and then the phthisical patient or the person predisposed to phthisis need not suffer.

The conclusion is arrived at that much of the improvement in the phthisis mortality which had seemed to Buchanan and others to be due to drying of the soil was really to be attributed to the coincident improvements in the social condition of the people in regard to housing and food which had taken place in the same period. Dr. Newsholme's view is that "personal infection is the main cause of the spread of phthisis, and that this occurs chiefly where people are most closely agglomerated, and live an indoor life; that deficient nutrition is an important favouring cause of phthisis, and that wetness of soil operates in a minor degree by favouring catarrhal conditions of the respiratory mucous membrane."

[1] Practitioner, Feb. 1901.

